# Goniometer Crosstalk Compensation for Knee Joint Applications

**DOI:** 10.3390/s101109994

**Published:** 2010-11-09

**Authors:** Tatiana de Oliveira Sato, Gert-Åke Hansson, Helenice Jane Cote Gil Coury

**Affiliations:** 1 Department of Physical Therapy, Universidade Federal de São Carlos, CP 676, CEP 13565-905, São Carlos, SP, Brazil; E-Mail: helenice@ufscar.br (H.J.C.G.C.); 2 Division of Occupational and Environmental Medicine, University Hospital, SE-22185, Lund, Sweden; E-Mail: gert-ake.hansson@med.lu.se (G.A.H.)

**Keywords:** knee, gait, movement, measurement errors

## Abstract

Electrogoniometers are prone to crosstalk errors related to endblocks rotation (general crosstalk) and to the characteristics of each sensor (individual crosstalk). The aim of this study was to assess the crosstalk errors due to endblock misalignments and to propose a procedure to compensate for these errors in knee applications. A precision jig was used to simulate pure ±100° flexion/extension movements. A goniometer was mounted with various degrees of valgus/varus (±20°) and rotation (±30°) misalignments. For valgus/varus misalignments, although offset compensation eliminated the error in the valgus/varus recordings for 0° of flexion/extension and reduced it to a few degrees for small (±30°) flexion/extension angles (root mean square error = 1.1°), the individual crosstalk caused pronounced errors for large (±100°) angles (18.8°). Subsequent compensation for this crosstalk reduced these errors to 0.8° and 4.5°, respectively. For rotational misalignment, compensation for the general crosstalk by means of coordinate system rotation, in combination with compensation for the individual crosstalk, reduced the errors for small (±30°) and large (±100°) flexion/extension angles from 3.6° to 0.5° and from 15.5° to 2.4°, respectively. Crosstalk errors were efficiently compensated by the procedures applied, which might be useful in preprocessing of knee functional data, thereby substantially improving goniometer accuracy.

## 1. Introduction

Direct measurements of joint angles can be conducted through flexible electrogoniometers, which have been used in clinical research [1,2]. For accurate results, the goniometers, as well as video and electro-optical recording systems [3,4], need to represent the planes of the movement to be recorded. If not, crosstalk is introduced, *i.e.*, a phenomenon in which movements performed exclusively in one plane are recorded as a false signal in the orthogonal planes.

Two main types of goniometer crosstalk can be identified: (a) the *general crosstalk*, when there is rotation between the two endblocks of the goniometer; (b) the *individual crosstalk*, which is introduced by the goniometer itself. For the general crosstalk, effective compensation can be achieved by recording the rotation, e.g., by means of a torsiometer, and use this rotation to perform the coordinate system rotation [5,6]. For the individual crosstalk, which may vary considerably for electrogoniometers of the same brand and type, a method for modeling and compensating for this crosstalk has recently been developed [7].

For the knee, flexion/extension is the dominant movement direction. However, the valgus/varus movements may be as clinically relevant as the flexion/extension, since they relate to joint stability. Increased valgus/varus movements might contribute towards the medial compartment degeneration often observed in patients with anterior cruciate ligament (ACL) deficiencies [8]. Hewett *et al.* [9] also demonstrated that increased valgus movement during the impact phase of jump-landing tasks is a key predictor of increased risk of ACL injuries in females. For assessing these clinically significant but, in relation to the flexion/extension movements, relatively small valgus/varus movements, measurement methods with small crosstalk errors are necessary.

An additional complication is that the knee joint present anatomically different configurations between subjects, e.g., frontal plane misalignments of the goniometers due to valgus (“knock kneed”) or varus (“bow legged”) posture. Moreover, musculoskeletal conditions, for instance due to differences between before and after training, may introduce goniometer alignment variations because of muscle hypertrophy after training [10].

**Figure 1.**
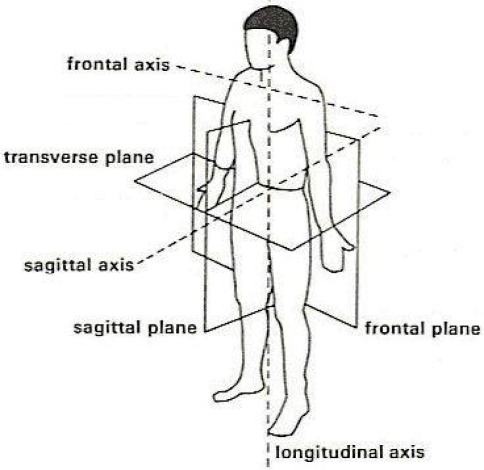
Body planes and axis. Frontal axis is defined along medio-lateral direction; sagittal axis is defined along antero-posterior direction and longitudinal axis is defined along superior-inferior direction.

**Figure 2.**
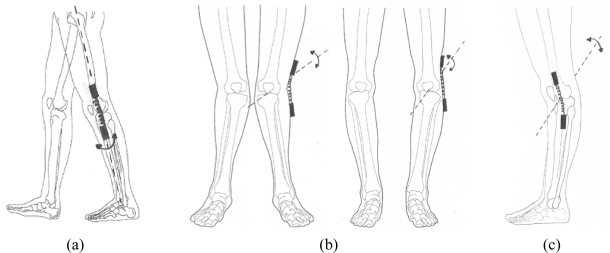
Types of knee endblock misalignments: **(a)** around the longitudinal axis (in the transverse plane); **(b)** around the sagittal axis (in the frontal plane); and **(c)** around the frontal axis (in the sagittal plane).

Three main types of deviation may occur when attaching the goniometer endblocks to the subject’s knee: (A) around the longitudinal axis, applying a rotation between the endblocks in the transverse plane; (B) around the sagittal axis, moving the two endblocks in the frontal plane, due to valgus or varus posture; and (C) around the frontal axis, moving the endblocks in the sagittal plane. Body planes and axis are presented in Figure 1 and its application for knee misalignments are shown in Figure 2.

The effect of misalignment around the longitudinal axis, *i.e.*, rotation between the endblocks of the goniometer [*cf.*, Figure 2(a)], can be compensated for, regarding both the general and the individual crosstalk, provided that the rotation angle between the endblocks and that the individual crosstalk of the goniometer are known. Since the algorithms for these compensations refer to the mechanical reference position of the goniometer, *i.e.*, the endblocks of the goniometer aligned in all three dimensions, the output from the goniometer in this position (the offset value) has to be recorded and subtracted from the subsequently recorded data before applying these algorithms. This procedure of referring the measurements to a reference position is often referred to as “setting the zero position”. Misalignment due to valgus/varus [*cf.*, Figure 2(b)] and flexion/extension deviations [*cf.*, Figure 2(c)] might be compensated for by “setting the zero position” when the subject is standing in the anatomical reference position. Of course, by definition, the compensations for the valgus/varus and flexion/extension angles are valid for the reference position of the knee. However, the position of the goniometer when it is attached to a subject’s knee deviates from its mechanical reference position, and therefore, it is obvious that the crosstalk compensations are not valid during flexion/extension movements if the mechanical reference position of the goniometer is not used for “setting the zero position”.

Thus, the objective of this study was to assess the errors due to endblock misalignments in the frontal and transverse planes, and to propose and evaluate a procedure to compensate for these errors, during applications with one dominant movement direction.

## 2. Experimental Section

### 2.1. Equipment, Data Acquisition and Processing

**Figure 3.**
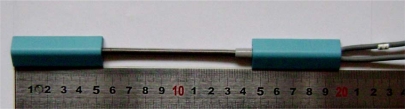
The biaxial goniometer (Biometrics Ltd., Gwent, UK; blue model [XM110]), used for knee recordings.

One biaxial electrogoniometer (M110, Biometrics Ltd., Gwent, UK) was evaluated (Figure 3). This sensor had previously been extensively used for knee angle recordings during gait, which increases the amount of individual crosstalk. The data were recorded at 100 Hz using an acquisition unit (DataLog, Biometrics Ltd., Gwent, UK). The electrogoniometer is composed by two plastic endblocks, which are connected by a spring; within this spring there are two pairs of thin resistor wires, orthogonally arranged along a flexible steel wire, constituting two strain gauges. The measurement is based on the deformation of the strain gauges, which affect the electric current passing through the resistor wires, which in turn is proportional to the angle between the two endblocks. One strain gauge records flexion/extension and the other valgus/varus, and the outputs are calibrated by the manufacturer. For details about the measurement principle of the goniometer see Rowe *et al.* [11]. For simulating pure flexion/extension angles, a precision jig was used. This jig was used in a previous study [7]. The lower segment, regarded as the shank, was fixed and jig motions were accomplished by moving the upper segment (regarded as the thigh). Backward motion of the upper segment thus represented flexion (positive values) and forward motion extension (negative values). The applied range of motion was 100° for both flexion and extension; this is relevant since extension 0–100° for the right knee corresponds to flexion 0–100° when the goniometer is attached to the left knee.

**Figure 4.**
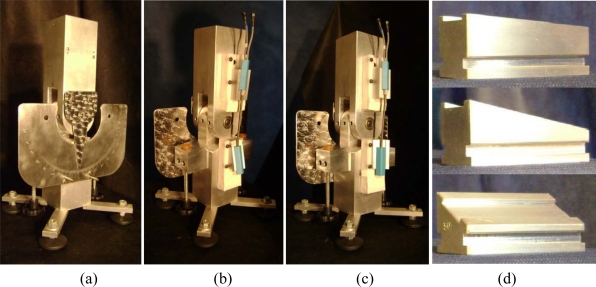
Jig, wedges and electrogoniometer used in the tests. **(a)** The graded angular scale is seen at the back of the jig; **(b)** 20° valgus misalignment wedges (in bright color) applied to the goniometer endblocks mounted in the jig: upper wedge 6° and lower wedge 14°;**(c)** 30° external rotational misalignment applied to the goniometer endblocks mounted in the jig: upper wedge 0° and lower wedge 30°; **(d)** 6° valgus/varus wedge (top), 14° valgus/varus wedge (middle) and 30° rotation wedge (bottom).

Wedges were used to simulate frontal and transverse plane misalignments. Endblock misalignments for valgus/varus were obtained by means of a pair of wedges, one at the thigh and the other at the shank, and for rotation by means of one wedge at the shank (Figure 4). Valgus endblock misalignment was defined as positive and varus as negative. Valgus misalignments of 20°, modeled as the sum of the misalignments of the endblocks on the thigh and the shank, were obtained as ten combinations (2°/18°, 4°/16°, …, 20°/0°; the first number representing the angle of the wedge on the thigh and the second one the angle of the wedge on the shank). The corresponding varus misalignments were obtained in an analogous manner.

The rotational misalignments were obtained by keeping the endblock on the thigh at a neutral angle (0°) and applying a rotation wedge to the shank. Rotational misalignments ranging from 2° to 30° with an increment of 2° were tested. Both internal (+) and external (−) rotation were evaluated giving 30 trials. These maximum frontal and transverse plane misalignments seemed to be reasonable and comparable to the total misalignment applied to the endblocks due to knee attachment [12]. No flexion/extension misalignment was applied. The data were processed using the DataLog PC Software version 3.0 (Biometrics Ltd., Gwent, UK) and a customized software developed using MatLab version 7.0.1 (MathWorks Inc., Natick, MA, USA).

### 2.2. Procedures

The first step in data collection was to “set the zero position” for both channels of the goniometer, *i.e.*, on a table and aligned to a ruler, which is a prerequisite for performing the compensations for the general and the individual crosstalk. The goniometer was then attached to the jig, above the wedges, to simulate misalignments of the goniometer endblocks.

For each trial of valgus/varus misalignment, two recordings were performed. The first recording was static with the jig in the zero position. This recording showed the effect of the frontal plane deviation on the valgus/varus recordings. The second recording was dynamic, with the thigh in continuous movement of amplitude ±100° of flexion/extension, for one minute, during which about 15 cycles were performed. For each of the 30 rotational misalignment trials, dynamic recording was performed. The maximum flexion/extension tested angles is sufficient to represent the knee angle during gait. Before conducting the main study, the individual crosstalk for the goniometer was recorded and modeled by a polynomial of degree 8 and an interval length of 5°; for details see Sato *et al.* [7].

### 2.3. Data Analysis

For each of the *valgus/varus misalignments*, the data were first compensated for the offset by subtracting the summed angles of the wedges, and then for the individual crosstalk as described by Sato *et al.* [7]. For *rotational misalignment*, the data were first compensated for the general crosstalk by performing a transformation of the flexion/extension valgus/varus outputs corresponding to a coordinate system rotation as described by Hansson *et al.* [6], and then for the individual crosstalk .

For *valgus/varus misalignments*, the recorded valgus/varus and flexion/extension angles were presented in X-Y plots, and the root mean square (RMS) values of the valgus/varus signal were calculated, for the raw, the offset compensated, as well as the offset and individual crosstalk compensated data. For *rotational misalignments*, the recorded valgus/varus and flexion/extension angles were presented in X-Y plots, and the RMS values of the valgus/varus signal were calculated, for the raw, the rotation compensated, as well as the rotation and individual crosstalk compensated data. RMS values provide a measure of the average variation of the measured angles, disregarding whether this variation was in a positive or negative direction. To evaluate errors over different ranges of movement, the data representing movements within the ranges of ±30° and ±60° and over the full range of ±100° were analyzed separately.

## 3. Results

**Figure 5.**
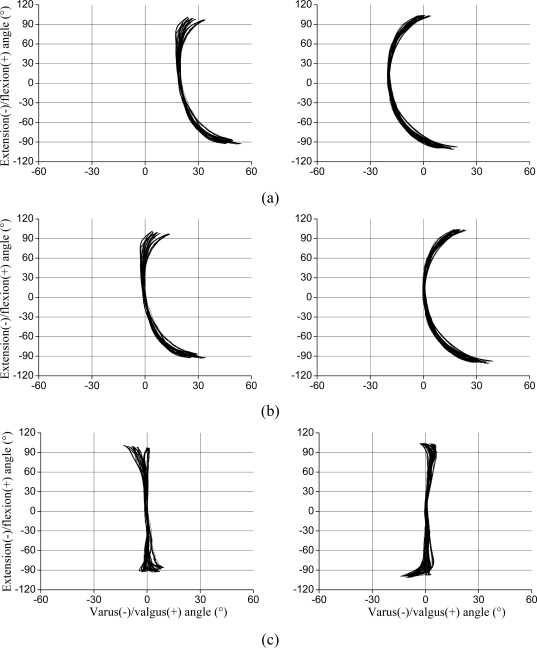
X-Y plots of valgus/varus and flexion/extension angles for valgus (left graphs) and varus (right graphs) misalignments of 20° distributed between the thigh and shank (10 trials). For each trial, about 15 movements of ±100° pure flexion/extension were performed. **(a)** Raw data; **(b)** Offset compensated data; **(c)** Offset and individual crosstalk compensated data.

Regarding valgus/varus misalignments, in the static 0° flexion/extension position of the jig, the recorded valgus/varus offset corresponded to the summed misalignment, independent of the distribution of the misalignment between the thigh and the shank. During the ±100° of flexion/extension movements, valgus/varus misalignment caused a corresponding shift in the valgus/varus angles over the whole range of motion [Figure 5(a)]. Hence, by subtracting the offset, it was possible to correct for this error, as shown in Figure 5(b). Although the offset compensation eliminated the error for 0° of flexion/extension and reduced it to a few degrees for small (±30°) flexion/extension angles (1.1°; Table 1), the individual crosstalk caused a pronounced error for angles ranging ±100° [18.8°; Figure 5(b) and Table 1]. By also compensating for the individual crosstalk, the error was further reduced, although this effect was still visible for angles exceeding 30° [Figure 5(c)]. For angles in the range ±60°, the error was reduced by a factor of 2 to 3. Nonetheless, the X-Y graphs for the fully compensated data [Figure 5(c)] present a curved pattern, particularly at flexion/extension angles close to 100°.

**Figure 6.**
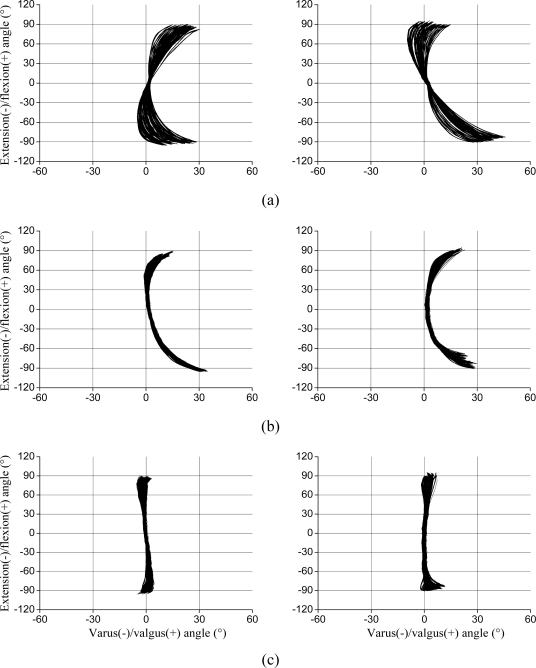
X-Y plots of valgus/varus and flexion/extension angles for internal rotation (left graphs) and external rotation (right graphs) misalignments from 2° to 30° in steps of 2° (15 trials). For each trial about 15 movements of ±100° pure flexion/extension were performed. (a) Raw data; (b) General crosstalk compensated data; (c) General and individual crosstalk compensated data.

Regarding rotational misalignments, the X-Y graphs show that the goniometer rotation was proportional to the rotation angle applied by the wedge [Figure 6(a)]. After rotating the coordinate system by half the angle of the wedge [6] the data revealed the individual crosstalk [Figure 6(b)]. The compensation for the individual crosstalk reduced the error considerably and the residual error is shown in Figure 6(c). The RMS error decreased after applying the compensation procedures, especially for low (±30°) flexion/extension amplitudes (RMS error = 0.7°; Table 1). It can also be seen in Table 1 that the maximum residual RMS error for the total (±100°) flexion/extension amplitude is 2.4°.

For some of the tested misalignments the recorded flexion/extension values were lower than the actual angles, *i.e*., there was a gain error in the flexion/extension recordings. For valgus misalignments the recorded flexion/extension was about −90° at −100° (Figure 5), and for rotation misalignments in both directions, the recorded flexion/extension was about ±90° for ±100° (Figure 6), *i.e*., the gain error was about 10%.

## 4. Discussion

Valgus/varus misalignments of ±20° could be compensated by offset subtraction. The distribution of the valgus/varus misalignment at the thigh and the shank did not interfere in the offset compensation. Subsequent individual crosstalk compensation provided an additional and, for flexion/extension angles above 60°, substantial reduction in the errors. Rotation misalignments of ±30° showed an evident crosstalk pattern, and compensation based on coordinate system rotation was an adequate first step. However, subsequent individual crosstalk compensation was required in order to obtain lower errors. The coordinate system rotation used here for compensation worked well for the situation that was tested, in which only the endblock on the shank was rotated.

Buchholz and Wellman [5] stated that rotation led to crosstalk errors because the strain gauges that defined the measurement axis might be rotated in relation to the plane of the movement. However, crosstalk effect can also occur when using video and electro-optical recording systems, due to the misalignment of the movement axis [3,4]. Moreover, crosstalk seems to affect particularly motion occurring in joints presenting one major component uniaxially, as the knee joint.

The goniometer compensations showed better results for flexion/extension angles up to 60°, which is the maximum knee flexion angle required in the swing phase of normal gait [11,13]. Functional activities that involve higher knee flexion angles, such as running, stair climbing and sitting down [14] might still be affected by residual crosstalk errors.

Regarding rotation compensation, the coordinate system used here for compensation worked well for the situation that was tested, in which only the endblock on the shank was rotated. Our setup in the jig made the presumption of correct alignment of the endblock on the thigh in the transverse plane. If this prerequisite was not fulfilled, *i.e*., if both endblocks were rotated, the basic assumption of alignment on the main plane of movement would be violated. Of course, it is not possible to compensate for this type of misalignment of the endblock on the thigh.

In the present study, the data for offset compensation of valgus/varus misalignment were derived from the angles of the precision wedges used in the trials. During the static tests of the jig, the difference between the wedges and recorded valgus/varus angles was very small. Hence, for recordings within clinical practice, the valgus/varus angle at anatomical reference position should be recorded, and these values should be used for the offset compensation. For rotational misalignment, the general crosstalk compensation was based on the angle of the precision wedges used. For measurements within clinical practice, the rotation between the endblocks can be measured using a torsiometer applied on top of the goniometer. One advantage of the torsiometer is that, besides recording the rotational misalignment at the anatomical reference position, the rotation of the tibia during gait is recorded and dynamic compensation for the general crosstalk can be applied; this approach has been applied to the wrist and forearm by Hansson *et al*. [6].

Whether the gain error for flexion/extension, which was about 10% for the full range of flexion/extension angles for some of the misalignments, is evenly distributed over the full range, or more pronounced at the extreme angles, cannot be determined in the present study. However, considering that the crosstalk errors, both the raw and the compensated ones, show their highest values at the extreme angles, also the gain errors might be higher for the extreme angles. Although the gain error in flexion/extension has to be considered, it does not contribute to the crosstalk error, which is the critical one for accurate recording of valgus/varus of the knee.

For high amplitude flexion/extension movements, the valgus/varus residual error, as well as the flexion/extension gain error, for the sensor used in this study was substantial. This is presumably due to the high individual crosstalk of this goniometer, which had already been used in previous studies. Although the compensation worked well for the present sensor, new sensors will presumably show smaller individual crosstalk, and thus also smaller residual and gain errors.

## 5. Conclusions

Compensation procedures for valgus/varus and internal/external rotation endblock misalignments, including offset and general and individual crosstalk compensation, reduced the crosstalk errors substantially. The prerequisites for using these compensation procedures are recording of the mechanical reference position and the rotational angles between the endblocks of the goniometer. In addition the individual crosstalk of the goniometer has to be known. These procedures were successful applied for preprocessing of data and substantially improved the accuracy of the movement recorded by goniometer.

## Figures and Tables

**Table 1.** Mean error in valgus/varus recordings for one goniometer mounted with valgus/varus and internal/external rotational misalignments of ±20° and ±30° respectively, during pure flexion/extension movements with ±100° amplitude. Root mean square (RMS) errors are shown for ±30°, ±60° and ±100° range of motion. For valgus/varus misalignments, RMS errors are shown for data without any compensation (none), with compensation for valgus/varus offset (offset) and with compensation for offset and individual crosstalk (offset and individual crosstalk). For internal/external rotational misalignments, RMS errors are shown for data without any compensation (none), with compensation for general crosstalk and with compensation for general and individual crosstalk (general and individual crosstalk).

**Misalignment**	**Compensation**	**Movement range (°)**		
		**± 30**	**± 60**	**± 100**
		*error (RMS; °)*		
Valgus; 20°	none	20.0	21.1	31.3
	offset	1.0	2.6	14.2
	offset and	0.6	0.8	3.6
	individual crosstalk			
Varus; 20°	none	19.4	18.1	13.5
	offset	1.1	3.1	18.8
	offset and	0.8	1.4	4.5
	individual crosstalk			
Internal rotation; 30°	none	2.5	4.7	11.7
	general crosstalk	2.1	3.9	12.3
	general and individual crosstalk	0.7	1.1	1.5
External rotation; 30°	none	3.6	6.8	15.5
	general crosstalk	2.3	3.9	11.6
	general and individual crosstalk	0.5	0.8	2.4
